# Is it possible ABC transporters genetic variants influence the outcomes of a weight-loss diet in obese women?

**DOI:** 10.1590/1678-4685-GMB-2019-0326

**Published:** 2020-07-31

**Authors:** Mayza Dalcin Teixeira, Luciane Viater Tureck, Gabrielle Araujo do Nascimento, Ricardo Lehtonen Rodrigues de Souza, Lupe Furtado-Alle

**Affiliations:** 1 Universidade Federal do Paraná, Departamento de Genética, Laboratório de Polimorfismos e Ligação, Curitiba, PR, Brazil.

**Keywords:** Cholesterol metabolism, obesity, genetic polymorphisms, dietary intervention, lipid profile

## Abstract

ATP-Binding Cassette (ABC) transporters are involved in cholesterol metabolism and their dysfunctions could lead to obesity-associated complications. It was investigated whether SNPs in the *ABCA1* (rs1800977 and rs2230806), *ABCA7* (rs2279796) and *ABCG1* (rs692383 and rs3827225) genes can modulate the responsiveness of 137 obese women to a weight-loss dietary intervention. Thus, anthropometric and lipid profiles were collected at baseline and after nine weeks of a calorie-restricted diet of 600kcal per day and participants were genotyped for the *ABC* genes SNPs. Regarding the transversal analysis, the *ABCA7* rs2279796 GG genotype was associated with higher levels of total cholesterol and LDL-c at baseline (p = 0.044 for both). Association between *ABCG1* rs692383 AA genotype and lower BMI were found in the post-diet moment, however, statistical significance was lost after multi-test correction. Regarding the longitudinal analysis, after multi-test correction, the association remained between *ABCG1* rs692383 G allele and HDL-c levels: G allele carriers had a lower HDL-c reduction (p = 0.043). Results suggest the standard weight-loss diet applied in this study could attenuate the *ABCA7* rs2279796 GG genotype effects found at baseline and non-dyslipidemic obese women with *ABCG1* rs692383 G allele are benefitting from the diet with a lower reduction in HDL-c levels.

## Introduction

Lipid homeostasis is an important protective factor against several diseases, such as obesity, dyslipidemia, cardiovascular diseases, type 2 diabetes and inflammation (Lee *et al.*, 2003). Correct functioning of many gene products is involved in the lipid homeostasis, among these genes are the ATP-Binding Cassette (ABC) transporters. The ABC transporters are cell membrane proteins which use the energy from ATP hydrolysis to transport molecules from inside or outside of cell (Wilkens, 2015). In general, these proteins transport many molecules, such as polysaccharides, proteins, vitamins, hormones, oligonucleotides and lipids (Kaminski *et al.*, 2000; Wilkens, 2015).

The ABCA1, ABCA7 and ABCG1 proteins perform cholesterol and phospholipids efflux from cells and are involved in the high-density lipoprotein (HDL) biogenesis (Takahashi *et al.*, 2005). Changes in protein expression and/or structure could alter lipid transportation leading to imbalanced lipid homeostasis and metabolic disturbances (Large *et al.*, 2004; Takahashi *et al.*, 2005; Porchay-Baldérelli *et al.*, 2009; Xu *et al.*, 2011). Consequently, the correct operation of ABC transporters is essential to maintain body homeostasis, especially regarding the cholesterol metabolism.

The *ABCA1* gene product is essential to HDL biogenesis by cholesterol and phospholipid efflux mediation to the free-lipid ApoA-I protein (Terasaka *et al.*, 2008). Hence, rare mutations in the *ABCA1* gene are related to monogenic hypercholesterolemia, as Tangier disease and hypoalphalipoproteinemia (FHA). Both diseases are characterized by low HDL-c plasmatic levels and an increased coronary artery disease risk (Bodzioch *et al.*, 1999; Genvigir *et al.*, 2008; Porchay-Baldérelli *et al.*, 2009).

ABCA7 is the ABC transporter with the highest homology with ABCA1 protein but it has a different tissue-specific expression profile (Abe-Dohmae *et al.*, 2006; Yang and Gelissen, 2017). The ABCA7 protein, as well as ABCA1, is related to HDL synthesis, but its absence does not alter significantly the HDL-c levels, neither cholesterol or phospholipids efflux or serum triglycerides and free fatty acids levels (Kim *et al.*, 2005; Abe-Dohmae *et al.*, 2006). Abe-Dohmae and colleagues hypothesized that ABCA7 mimics the ABCA1 function, but it is not essential as ABCA1 to the HDL enrichment.

The ABCG1 protein is predominantly localized within intracellular membranes and acts in a way similar to ABCA1 protein. After cholesterol loading, endosomes containing ABCG1 are mobilized to the cell membrane and, differently of the ABCA1, perform the cholesterol efflux to HDL particles without interacting with an extracellular protein, such as ApoA-I (Tarling and Edwards, 2011; Cheng *et al.*, 2016). Moreover, the ABCG1 protein differs from ABCA1 because it requires phospholipids-rich cholesterol receptors (Sankaranarayanan *et al.*, 2009). Consequently, ABCA1 and ABCG1 have related functions and work synergistically. ABCA1 is responsible for the lipid enrichment of the lipid-free ApoA-I and generates a receptor to ABCG1-mediated cholesterol efflux (Gelissen *et al.*, 2006; Out *et al.*, 2008).

Adipose tissue performs a main role in lipid homeostasis, because it is the largest pool of cholesterol in the body (Yu *et al.*, 2010). Since the ABCA1, ABCA7 and ABCG1 proteins act mainly in the cholesterol metabolism, and cholesterol dysfunction in the adipose tissue could be related to a lipid imbalance in the whole body, genetic polymorphisms in the corresponding genes could be involved in obesity-associated metabolic complications. However, *ABC* genes polymoprhism effects in serum lipid levels and anthropometric measurements in obesity are not completely understood. Moreover, the interaction of *ABCA1*, *ABCA7* and *ABCG1* genetic variants with the responsiveness to a dietary intervention which aims weight loss in obese individuals has not been investigated yet.

Thus, the aim of this study was to verify whether single nucleotide polymorphisms (SNPs) in the *ABCA1*, *ABCA7* and *ABCG1* genes influence variables related to obesity and lipid profile in obese women and to evaluate the effect of these SNPs on anthropometric and lipid parameters in response to a weight-loss dietary intervention. We have hypothesized that some of the *ABCA1*, *ABCA7* and *ABCG1* genotypes would be associated with a low response to the weight loss dietary intervention. In other words, a general weight loss diet might not benefit all obese individuals equally.

## Material and Methods

### Overview of the study

Participants enrolled in this study are part of a broader project in nutrigenetics, conducted by our research group. The socioeconomic characteristics of the participants and the dietary outcomes regarding weight-related variables were previously published by Saliba *et al.* (2014) and Nascimento *et al.* (2017), respectively. The present study focused on the lipid profile and related genes.

**Figure 1:**
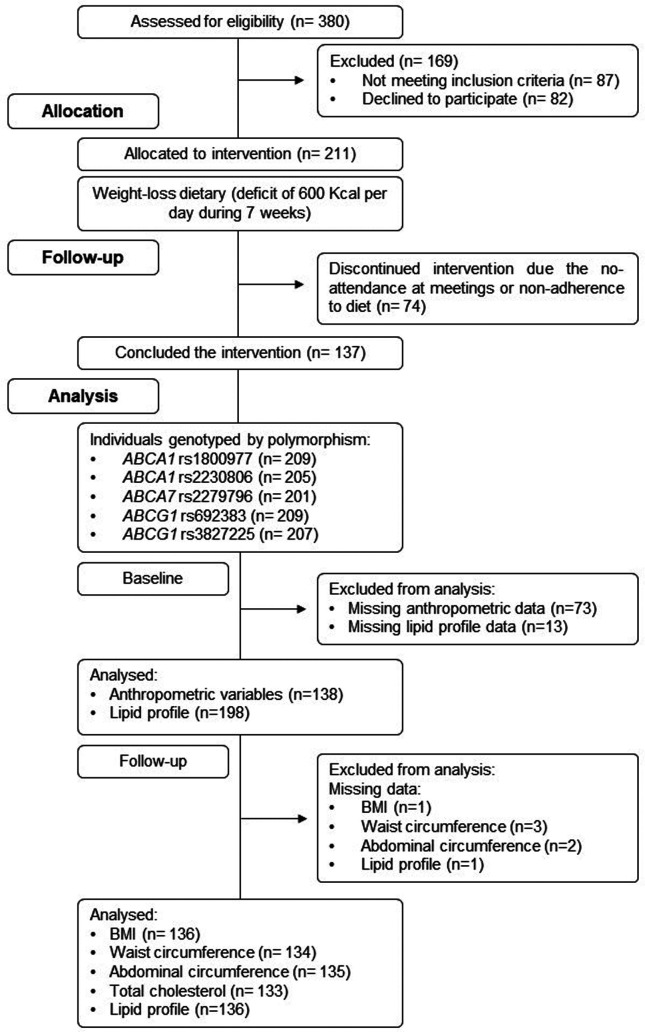
Consort diagram of the study design and flow.

The initial sample, with all individuals who met the inclusion criteria, was composed of 211 obese women with predominantly European ancestry (self-declared); of which 134 completed the dietary intervention program during nine weeks in the city of Curitiba, Paraná State, Brazil (Figure 1).

The study design was longitudinal, thus lipid profile and anthropometric data were measured at baseline and after the dietary intervention program. Participants were genotyped for *ABCA1*, *ABCA7* and *ABCG1* gene polymorphisms, and associations between polymorphisms and continuous variables were investigated by multivariable models. The longitudinal analysis consisted in the investigation of the effect of the dietary intervention program, and were based on the variation between baseline and after-diet measures. Moreover, a transversal analysis was performed in both moments of the study: at baseline and after dietary intervention.

### Subjects

Participants were invited to participate in the study by local radio and television. The inclusion criteria were: to be female, to be obese (BMI [#GTEQ#] 30 Kg/m^2^), to be 20 years old or older. Pregnant, breastfeeding and climacteric participants were excluded from the study, as well as, women with hypothyroidism, type I diabetes, kidney diseases, hypertension, in medicinal drug treatment for weight control and that have been through stomach reduction surgery.

The participants who met the inclusion criteria were informed about the study and the informed consent was obtained from all subjects. This research was approved by the ethics committee of the Pontifical Catholic University of Paraná’s Institutional Ethics Board (IEB approval number: 0005306/11) (Saliba *et al.*, 2014).

### Dietary intervention program

The dietary intervention program lasted nine weeks and consisted of two weeks of a pre-intervention period and seven weeks of a dietary intervention for weight loss. Initially, 211 obese women were submitted to the program.

### Pre-intervention (2 weeks)

Personalized diets with a deficit of 600 kcal based on the consumption preferences and individual total energy expenditure were elaborated in this phase. Moreover, anthropometric data (height, weight, waist and abdominal circumferences) was collected, as well as peripheral blood for genetic analyses and lipid profile measure during fasting.

Resting metabolic rate was calculated for each participant according to the formula: [10 x weight (kg)] x [6.25 x height (cm)] – [5 x age (years)] – 161. Then, it was calculated individual total energy expenditure, according to the formula: [resting metabolic rate x 1.3)] (Seagle *et al.*, 2009). Each participant carried out a 24-hour dietary recall about the details of food and drink consumption in the previous 24h to identify individual dietary habits and preferences. Participants reported the number and types of meals consumed in the last 24 h, how they usually prepare the meals, frequency of consumption and intake of food and treats, as well as the intake of water and alcohol.

Then, based on the individual preferences, the total energy expenditure and the dietary guidelines for weight loss (described below), a nutricionist designed an individual diet for each participant, whose total amount of calories was calculated by the formula: [total energy expenditure (Kcal) – 600], generating diets with a deficit of 600 kcal regarding the daily energy expenditure. Thus, the diets ranged from 1000 to 2200 kcal per day. The deficit of 600 kcal was based on the American Dietetic Association that states a reduction in the energy intake of 500 to 1,000 kcal/day is necessary to a 0.5 to 1kg of weight loss per week (Seagle *et al.*, 2009).

In general, individual diets were based on a standard model of diet to non-dyslipidemic individuals, in which the energy consumption ranges were 45% to 65% from carbohydrates, 20% to 35% from fats and 10% to 35% from proteins. The energy consumption was distributed in four meals per day (breakfast, lunch, afternoon snack and dinner). The latter (dinner) was rich in salad/vegetables, moderate in protein (chicken, meat or cheese) and with low quantities of carbohydrates (rice or bread), thus, two general options for dinner were recommended: (1) salad/vegetables, bread and cheese or (2) and salad/vegetables, rice, beans and chicken (or meat). Products which are commonly found in Brazilian meals were added to the diets, such as bread, rice, beans, coffee, soy oil, margarine and oats. This general diet model aimed to standardize the individual diets regarding macronutrients, calcium and iron quantities and to contemplate the Brazilian National recommendations of healthy eating habits (Brasil, 2008).

### Dietary intervention for weight loss (7 weeks)

During this phase, besides the application of the diet itself, three meetings were held: the first one was an individual orientation one month after the beginning of the diet. This meeting focused in clarifying doubts about the dietary program and checking the food intake diary, which contained the records of daily consumption of food and drink of each participant. The two other meetings occurred in groups, as following: the first meeting was a lecture about the importance of choosing healthy foods and how to make healthy food substitutions. The second meeting was a workshop about the interpretation of food labels.

At the end of the program, 74 obese women did not fully adhere to the diet or discontinued the intervention. Thus, 137 obese women concluded the dietary intervention program, and their anthropometric data and lipid profile were measured again (Figure 1).

### Anthropometric and biochemical variables

The anthropometric variables analyzed were weight (measured without shoes and wearing light clothes, kg), body mass index (BMI, calculated by the formula: [weight (kg)/height2 (m)], kg/m^2^), waist circumference (WC, cm) and abdominal circumference (AC, cm). WC was measured during expiration, at the mid point between the lowest rib and the iliac crest, and AC was measured from the high point of iliac crest during minimal breathing (Lohman *et al.*, 1988).

The biochemical variables analyzed were triglycerides (TG), total cholesterol (TC), low-density lipoprotein cholesterol (LDL-c), high-density lipoprotein cholesterol (HDL-c) and very low-density lipoprotein (VLDL) serum levels (all mg/dL). HDL-c and TG levels were measured by colorimetric method, and TC levels were measured enzymatically. LDL-c levels were determined by the Friedewald equation (Friedewald *et al.*, 1972), and VLDL levels were calculated by the equation: VLDL= [TG/5].

### Genetic data

The *ABC* genes polymorphisms investigated in this study are described in Table 1. We selected single nucleotide polymorphisms which have the minor allele frequency (MAF) higher than 10% in the CEU population (European ancestry) and present no linkage disequilibrium between themselves. Moreover, the following additional criteria were used: (1) polymorphisms which showed association with lipid profile and/or obesity-related traits in previous studies; (2) polymorphisms which showed potential functional or regulatory role.

**Table 1:** *ABC* genes polymorphisms investigated in this study.

Gene	Polymorphism	Assay ID	Location ^a^	Position ^a^	Alleles	MAF ^a^	Polymorphism effect
*ABCA1*	rs1800977 (C69T)	C___9456257_10	5’ prime UTR	9:107690450	G/A	0.318 (A)	A allele associated with higher *ABCA1* gene expression ^b. c^
*ABCA1*	rs2230806 (R219K)	C___2741051_1_	Exon 7 (missense)	9:107620867	C/T	0.207 (T)	The substitution is in the first extracellular loop, in a region that interacts with ApoA-I protein
*ABCA7*	rs2279796	C__15968630_10	Intron	19:1059004	G/A	0.414 (G)	G allele associated with higher *ABCA7* gene expression in the whole blood ^d^
*ABCG1*	rs692383	C___2947288_20	Intron	21:43635174	G/A	0.242 (G)	Unknown
*ABCG1*	rs3827225	C___1723781_30	Intron	21:43650779	G/A	0.278 (A)	Unknown

Notes: UTR: untranslated region. MAF: minor allele frequency of CEU individuals (European ancestry). ^a^ Ensembl database (Zerbino *et al.*, 2018); ^b^Hodoglugil *et al.* (2005); ^c^Porchay *et al.* (2006); ^d^ GTEx Portal (Lonsdale *et al.*, 2013).

Peripheral blood (10 mL) was collected in tubes with EDTA for each participant and the DNA was extracted according to the salting-out technique (Lahiri and Nurnberger, 1991). After DNA extraction, the samples were diluted to 20 ng/μl and were stored for posterior genotyping.

Participants were genotyped by TaqMan® allelic discrimination assay (Applied Biosystems, Life Technologies). Reactions were done according to the following conditions: 60 °C for 30 s and 95 °C for 10 min, 50 cycles of 95 °C for 15 s and 60 °C for 1 min, and 60 ºC for 30 s. Three previously sequenced control samples, representative of each of the possible genotypes, were included in each assay. In addition, in all assays was included a negative control (no DNA was added).

### Statistical analyses

Continuous variables were tested for normality by Kolmogorov-Smirnov test with Lilliefors correction. All variables showed a non-normal distribution. Thus, baseline and after-diet measures were compared by the Wilcoxon test, to verify the effect of the diet without considering genotypes.

Allele and genotype frequencies were obtained by direct counting, and Hardy-Weinberg equilibrium was verified by the chi-square test. The allele frequencies standard error was calculated according to the equation: SE= [(p frequency x q frequency)/(2 x total n)]^0,5^. Recessive, dominant and absence of dominance models of allelic interaction were tested for each SNP and the best-fitted model for our data was selected and was used in the following analyses. The dominant effect was assumed when the homozygous and heterozygous carriers showed similar phenotypes and this specific phenotype was different from the other type of homozygous. The recessive effect was assumed when only one type of homozygous showed a distinct phenotype, and the absence of dominance model was adopted when distinct phenotypes were observed in each genotype. The G allele dominant model was the best fit for *ABCG1* SNP rs692383 (AA x AG + GG); the G allele recessive model was the best fit for *ABCA1* SNP rs1800977 and *ABCG1* SNP rs3827225 (AA + AG x GG for both); the C and T alleles absence of dominance model was the best fit for *ABCA1* SNP rs2230806 (CC x CT x TT) and the A and G alleles absence of dominance model was the best fit for *ABCA7* SNP rs2279796 (AA x AG x GG).

Longitudinal and transversal associations of *ABC* SNPs with continuous anthropometric and biochemical variables were evaluated by general linear models (GLM). The outcome variables (BMI, WC, AC, TC, HDL-c, TG, LDL-c and VLDL) were tested with all the five SNPs in a multivariable model and were corrected by BMI (except when BMI was the outcome variable). Analyses were conducted in R software (R Core Team, 2018) and the statistical significance adopted for the tests was 0.05 (95% of confidence).

## Results

About 49% of the participants presented age between 30 and 39 years, 59% had a high socioeconomic level, 70% had a paid job, and 80% were not enrolled in school/college (Saliba *et al.*, 2014).

The dietary intervention resulted in reduction of weight, BMI, waist circumference, abdominal circumference, total cholesterol and HDL-c (p < 0.01 for all) (Table 2). The median (interquartile range) of reduction for these variables was: 2.30 (3.00) kg of weight, 0.85 (1.17) kg/m^2^ in the BMI, 4.00 (4.40) cm in the waist circumference, 7.00 (5.90) cm in the abdominal circumference, 5.00 (23.75) mg/dL in the total cholesterol and 4.00 (7.00) mg/dL in the HDL-c levels. The total cholesterol levels distribution is presented in Figure S1, where it is possible to see why the baseline and after diet medians are so close while the median of the difference between these two moments are negative, indicating a reduction in total cholesterol levels.

**Table 2:** Characteristics at baseline, after-diet moment and the variation in response to diet (median [IQR]).

Characteristic	N	Baseline	After diet	Variation	*P* value
Weight (Kg)	137	88.00 [15.50]	85.00 [16.70]	-2.30 [3.00]	< 0.0001
BMI (Kg/m^2^)	136	33.91 [6.69]	32.96 [6.97]	-0.85 [1.17]	< 0.0001
WC (cm)	134	93.87 [14.01]	89.50 [14.00]	-4.00 [4.40]	< 0.0001
AC (cm)	135	107.00 [15.90]	100.00 [17.00]	-7.00 [5.90]	< 0.0001
TC (mg/dL)	136	187.00 [49.50]	188.00 [49.50]	-5.00 [23.75]	0.008
HDL-c (mg/dL)	136	50.00 [14.00]	46.00 [13.00]	-4.00 [7.00]	< 0.0001
TG (mg/dL)	136	131.00 [68.25]	125.50 [80.25]	-1.00 [40.00]	0.990
LDL-c (mg/dL)	136	110.20 [36.10]	110.10 [43.60]	-1.40 [18.65]	0.527
VLDL (mg/dL)	136	26.20 [13.65]	25.10 [16.05]	-0.20 [8.00]	0.989

Notes: IQR: interquartile range; BMI: body mass index; WC: waist circumference; AC: abdominal circumference; TC: total cholesterol; TG: triglycerides. Variation was calculated by individual after - baseline values. P-value obtained from Wilcoxon test.

All the polymorphisms, except *ABCA1* SNP rs2230806, were in Hardy-Weinberg equilibrium (p > 0.05). The allele and genotype frequencies are shown in Table S1.

We evaluated polymoprhism associations with continuous variables at baseline (Table 3) and after dietary intervention (Table 4) (transversal analysis) by multivariable models. In addition, we investigated polymorphisms associations with the response to the weight-loss intervention by testing multivariable models using the variation between these two moments (longitudinal analysis) (Table 5). The multivariable models tested included all the five SNPs and were corrected by BMI (except when BMI was the outcome variable).

**Table 3:** Baseline characteristics (median [IQR]) according to the *ABCA1*, *ABCA7* and *ABCG1* genotypes.

Gene/SNP	Genotype	BMI (kg/m^2^)	WC (cm)	AC (cm)	TC (mg/dL)	HDL-c (mg/dL)	TG (mg/dL)	LDL-c (mg/dL)	VLDL (mg/dL)
*ABCA1* rs1800977	AA + AG	34.10 [6.55]	95.00 [13.60]	107.15 [14.17]	185.00 [47.00]	49.00 [14.00]	125.00 [74.00]	104.40 [30.60]	25.00 [14.80]
	GG	33.99 [6.20]	93.12 [14.29]	106.60 [15.10]	187.00 [47.00]	51.00 [13.00]	130.00 [60.00]	108.80 [38.40]	26.00 [12.00]
	*P* value ^a^	0.704	0.960	0.165	0.417	0.540	0.859	0.434	0.859
	*P* value ^b^	0.960	0.960	0.960	0.960	0.960	0.960	0.960	0.960
*ABCA1* rs2230806	CC	33.50 [7.12]	93.50 [12.89]	107.35 [15.10]	187.00 [51.00]	49.00 [13.00]	130.00 [68.00]	109.80 [36.40]	26.00 [13.60]
	CT	33.02 [4.77]	93.55 [10.67]	106.40 [12.47]	180.00 [39.50]	48.00 [14.00]	130.00 [64.00]	106.40 [33.40]	26.00 [12.80]
	TT	35.75 [6.34]	95.65 [12.62]	110.20 [15.70]	184.00 [45.00]	52.00 [15.75]	120.50 [72.25]	103.20 [35.25]	24.10 [14.45]
	*P* value ^a^	0.710	0.799	0.948	0.530	0.372	0.915	0.256	0.915
	*P* value ^b^	0.948	0.948	0.948	0.948	0.948	0.948	0.948	0.948
*ABCA7* rs2279796	AA	33.83 [6.67]	93.00 [10.60]	105.00 [12.00]	174.50 [36.75]	48.00 [16.50]	108.00 [75.50]	98.20 [28.80]	21.60 [15.10]
	AG	33.95 [6.52]	95.50 [12.70]	108.20 [15.55]	183.00 [44.00]	50.00 [12.00]	132.00 [67.50]	101.00 [31.20]	26.40 [13.50]
	GG	34.57 [7.98]	96.60 [18.80]	107.70 [21.25]	199.00 [47.00]	50.00 [15.00]	123.00 [63.00]	117.40 [31.40]	24.60 [12.60]
	*P* value ^a^	0.449	0.434	0.842	0.011	0.784	0.379	0.0089	0.379
	*P* value ^b^	0.599	0.599	0.842	0.044	0.842	0.599	0.044	0.599
*ABCG1* rs692383	AA	32.71 [5.15]	93.30 [11.47]	105.25 [14.67]	190.00 [47.75]	50.00 [13.75]	134.00 [59.00]	107.40 [37.70]	26.80 [11.80]
	AG + GG	34.51 [7.22]	94.80 [14.74]	108.15 [16.02]	182.50 [47.25]	50.00 [8.00]	119.00 [67.50]	106.40 [33.05]	23.80 [13.50]
	*P* value ^a^	0.052	0.461	0.226	0.814	0.909	0.042	0.550	0.042
	*P* value ^b^	0.139	0.733	0.452	0.909	0.909	0.139	0.733	0.139
*ABCG1* rs3827225	AA + AG	34.78 [6.89]	94.60 [13.60]	107.20 [17.10]	185.00 [51.00]	50.00 [12.00]	124.00 [71.50]	106.40 [36.40]	24.80 [14.30]
	GG	33.81 [6.30]	94.45 [14.35]	106.95 [14.00]	186.00 [46.00]	49.00 [15.00]	130.00 [61.00]	107.20 [35.60]	26.00 [12.20]
	*P* value ^a^	0.572	0.708	0.0328	0.618	0.330	0.894	0.317	0.894
	*P* value ^b^	0.894	0.894	0.262	0.894	0.88	0.894	0.88	0.894

Notes: IQR: interquartile range; BMI: body mass index; WC: waist circumference; AC: abdominal circumference; TC: total cholesterol; TG: triglycerides. ^a^*P* values for association between the *ABC* SNPs and continuous variables in multivariable models were obtained by using general linear model (GLM) adjusted by BMI, when possible. ^b^*P* values corrected for multiple testing.

**Table 4:** After dietary intervention characteristics (median [IQR]) according to the *ABCA1*, *ABCA7* and *ABCG1* genotypes.

Gene/SNP	Genotype	BMI (kg/m^2^)	WC (cm)	AC (cm)	TC (mg/dL)	HDL-c (mg/dL)	TG (mg/dL)	LDL-c (mg/dL)	VLDL (mg/dL)
*ABCA1* rs1800977	AA + AG	32.98 [6.76]	91.25 [14.32]	102.30 [16.30]	190.00 [45.25]	46.00 [13.00]	114.50 [79.50]	113.60 [42.80]	22.90 [15.90]
	GG	32.40 [6.78]	88.50 [15.50]	99.00 [18.00]	177.00 [46.50]	45.50 [13.00]	130.50 [72.50]	102.40 [35.70]	26.10 [14.50]
	*P* value ^a^	0.928	0.869	0.812	0.117	0.273	0.979	0.166	0.979
	*P* value ^b^	0.979	0.979	0.979	0.664	0.728	0.979	0.664	0.979
*ABCA1* rs2230806	CC	32.96 [6.76]	90.75 [13.55]	101.50 [8.50]	191.00 [50.50]	44.50 [10.75]	122.00 [86.25]	117.70 [49.60]	24.40 [17.25]
	CT	32.22 [4.56]	88.50 [14.00]	99.00 [13.60]	188.00 [27.00]	50.00 [15.00]	127.00 [77.00]	108.20 [39.20]	25.40 [15.40]
	TT	34.75 [7.07]	92.00 [14.40]	103.00 [16.20]	178.00 [59.00]	44.00 [13.50]	117.00 [58.50]	99.80 [33.90]	23.40 [11.70]
	*P* value ^a^	0.869	0.335	0.265	0.313	0.846	0.73	0.297	0.730
	*P* value ^b^	0.869	0.670	0.670	0.670	0.869	0.869	0.670	0.869
*ABCA7* rs2279796	AA	32.74 [7.45]	89.00 [15.20]	99.60 [12.80]	179.00 [38.00]	44.00 [14.00]	109.00 [53.00]	100.60 [36.40]	21.80 [10.60]
	AG	33.13 [6.01]	91.25 [13.00]	102.30 [16.30]	186.00 [51.00]	47.00 [11.00]	128.00 [76.00]	111.40 [44.80]	25.60 [15.20]
	GG	32.97 [6.01]	94.55 [17.72]	102.20 [22.25]	194.00 [42.00]	44.00 [13.00]	129.00 [95.00]	118.20 [36.00]	25.80 [19.00]
	*P* value ^a^	0.399	0.974	0.890	0.052	0.712	0.434	0.089	0.435
	*P* value ^b^	0.696	0.974	0.974	0.356	0.949	0.696	0.356	0.696
*ABCG1* rs692383	AA	32.33 [4.97]	88.00 [15.00]	96.20 [16.32]	189.00 [51.00]	43.00 [12.00]	129.00 [75.00]	105.40 [47.40]	25.80 [15.00]
	AG + GG	33.64 [7.06]	92.00 [13.92]	102.95 [16.22]	186.00 [46.50]	46.00 [13.50]	117.00 [76.00]	111.20 [40.30]	23.40 [15.20]
	*P* value ^a^	0.045	0.457	0.675	0.882	0.179	0.110	0.585	0.110
	*P* value ^b^	0.293	0.731	0.771	0.882	0.358	0.293	0.771	0.293
*ABCG1* rs3827225	AA + AG	33.91 [7.07]	91.00 [15.00]	103.00 [17.00]	188.00 [49.50]	44.00 [14.00]	130.50 [97.25]	107.20 [39.20]	26.10 [19.45]
	GG	32.46 [6.49]	90.50 [13.60]	100.00 [16.42]	188.00 [45.00]	47.00 [12.00]	119.00 [73.00]	113.20 [46.40]	23.80 [14.60]
	*P* value ^a^	0.449	0.213	0.966	0.837	0.509	0.484	0.921	0.484
	*P* value ^b^	0.814	0.814	0.966	0.966	0.814	0.814	0.966	0.814

Notes: IQR: interquartile range; BMI: body mass index; WC: waist circumference; AC: abdominal circumference; TC: total cholesterol; TG: triglycerides. ^a^*P* values for association between the *ABC* SNPs and continuous variables in multivariable models were obtained by using general linear model (GLM) adjusted by BMI, when possible. ^b^*P* values corrected for multiple testing.

**Table 5:** Characteristics changes after dietary intervention relative to baseline measures (median [IQR]) according to the *ABCA1*, *ABCA7* and *ABCG1* genotypes.

Gene/SNP	Genotype	BMI (kg/m^2^)	WC (cm)	AC (cm)	TC (mg/dL)	HDL-c (mg/dL)	TG (mg/dL)	LDL-c (mg/dL)	VLDL (mg/dL)
*ABCA1* rs1800977	AA + AG	-0.66 [1.15]	-3.95 [4.45]	-7.00 [6.23]	-4.00 [21.50]	-4.00 [6.00]	-2.00 [43.00]	-1.20 [13.90]	-0.40 [8.60]
	GG	-1.21 [1.16]	-4.05 [3.25]	-6.70 [5.70]	-5.00 [25.50]	-3.00 [7.50]	1.00 [42.00]	-2.60 [26.50]	0.20 [8.40]
	*P* value ^a^	0.019	0.893	0.146	0.972	0.849	0.709	0.863	0.709
	*P* value ^b^	0.152	0.972	0.584	0.972	0.972	0.972	0.972	0.972
*ABCA1* rs2230806	CC	-0.73 [1.38]	-4.00 [4.60]	-7.35 [-4.17]	-4.00 [24.00]	-4.00 [7.00]	1.00 [45.00]	-1.60 [20.20]	0.20 [9.00]
	CT	-0.86 [0.88]	-3.60 [3.75]	-5.55 [5.30]	-4.50 [22.75]	-4.00 [5.25]	-2.50 [39.50]	0.30 [20.85]	-0.50 [7.90]
	TT	-1.14 [1.21]	-4.80 [4.05]	-7.20 [5.30]	-5.00 [21.00]	-4.00 [8.00]	-7.00 [41.00]	-1.80 [12.20]	-1.40 [8.20]
	*P* value ^a^	0.336	0.023	0.152	0.356	0.189	0.507	0.846	0.507
	*P* value ^b^	0.569	0.184	0.504	0.569	0.504	0.579	0.846	0.579
*ABCA7* rs2279796	AA	-1.18 [0.60]	-4.00 [3.70]	-6.55 [5.12]	-5.00 [24.00]	-4.00 [5.00]	-12.00 [31.00]	1.20 [17.40]	-2.40 [6.20]
	AG	-0.74 [1.13]	-3.50 [4.60]	-7.40 [7.37]	-5.00 [22.50]	-3.00 [6.50]	1.00 [33.50]	-1.20 [16.30]	0.20 [6.70]
	GG	-0.90 [1.69]	-4.77 [3.92]	-6.85 [5.92]	-4.00 [25.00]	-5.00 [10.00]	5.00 [57.00]	-4.00 [25.60]	1.00 [11.40]
	*P* value ^a^	0.695	0.348	0.736	0.480	0.903	0.794	0.321	0.794
	*P* value ^b^	0.903	0.903	0.903	0.903	0.903	0.903	0.903	0.903
*ABCG1* rs692383	AA	-0.84 [0.93]	-3.95 [4.74]	-8.50 [5.30]	-9.00 [31.00]	-6.00 [8.00]	-12.00 [56.00]	-4.00 [23.40]	-2.40 [11.20]
	AG + GG	-0.83 [1.40]	-4.00 [3.60]	-6.30 [6.10]	-4.00 [20.00]	-3.00 [6.00]	1.00 [30.00]	-4.00 [17.00]	0.20 [6.00]
	*P* value ^a^	0.785	0.632	0.150	0.613	0.005	0.808	0.900	0.808
	*P* value ^b^	0.900	0.900	0.600	0.900	0.043	0.900	0.900	0.900
*ABCG1* rs3827225	AA + AG	-0.84 [1.20]	-4.75 [3.70]	-8.20 [5.40]	-5.00 [21.50]	-4.00 [7.00]	2.00 [49.00]	-4.50 [19.00]	0.4 [9.8]
	GG	-0.83 [1.24]	-3.50 [4.22]	-6.65 [6.50]	-4.00 [23.00]	-4.00 [7.00]	-4.00 [35.00]	0.80 [22.60]	-0.7 [6.9]
	*P* value ^a^	0.353	0.079	0.017	0.577	0.472	0.550	0.240	0.55
	*P* value ^b^	0.577	0.316	0.136	0.577	0.577	0.577	0.577	0.577

Notes: IQR: interquartile range; BMI: body mass index; WC: waist circumference; AC: abdominal circumference; TC: total cholesterol; TG: triglycerides. ^a^*P* values for association between the *ABC* SNPs and continuous variables in multivariable models were obtained by using general linear model (GLM) adjusted by BMI, when possible. ^b^*P* values corrected for multiple testing.

In the multivariable analysis of baseline measurements, we found the *ABCA7* SNP rs2279796 (GG genotype) association with higher levels of total cholesterol and LDL-c (p = 0.044 corrected for multiple tests for both) (Table 3). In addition, *ABCG1* SNP rs692383 AA genotype was associated with higher triglycerides and VLDL levels (p = 0.042 for both), and A allele of the *ABCG1* SNP rs3827225 was associated with a larger abdominal circumference (p = 0.0328) (Table 3). However, these associations lost significance after multi-test correction.

In the after-diet moment, multivariable analysis revealed an association between *ABCG1* SNP rs692383 G allele and a higher BMI (p = 0.045), which was lost after multi-test correction. No other association was found in the after-diet moment (Table 4).

Regarding the response of the variables to the diet, other associations were found, as shown in Table 5. *ABCA1* SNP rs1800977 GG genotype was associated with a higher BMI reduction in response to the diet (p = 0.019), and *ABCA1* SNP rs2230806 CC genotype was associated with a higher waist circumference reduction (p = 0.023). Both associations were lost after multi-test correction.

Moreover, *ABCG1* SNP rs3827225 A allele was associated with a higher abdominal circumference reduction (p = 0.017), but it was lost after correction. On the other hand, *ABCG1* SNP rs692383 G allele was associated with a lower reduction in the HDL-c levels (p = 0.043 corrected for multiple tests).

## Discussion

In this study, we evaluated the influence of five SNPs in *ABC* transporter genes on changes in anthropometric and lipid profiles of obese women who were submitted to a caloric restriction diet. Multiple associations between the SNPs and the investigated variables were identified, both with the initial and final measures, as well as with the variation between these moments.

Independent of the genotypes, the weight-loss dietary intervention contributed to a reduction in anthropometric measures, reflecting in positive outcomes to general health status. BMI, waist and abdominal circumferences reduced in response to the diet and the importance of these outcomes for women was discussed by Nascimento *et al.* (2017). On the other hand, the positive diet outcomes observed in anthropometrics were not observed in the lipid profile. The reduction of total cholesterol levels, which could be interpreted as a positive diet outcome, possibly was a result of the HDL-c decline after diet.

According to Bays *et al.* (2013), a high carbohydrate/low-fat diet usually leads to a reduction in HDL-c levels during the weight loss phase, and an increase in HDL-c levels usually occurs with weight stabilization. On the other hand, high fat and low carbohydrate/protein diets are healthier options to dyslipidemic patients because it tends to have a milder effect in HDL-c levels (Bays *et al.*, 2013). Considering that our sample was composed mainly by obese (BMI [#GTEQ#] 30) women who were not dyslipidemic, the general diet model applied in this study focused on the reduction of weight, and thus it was composed by percentages of carbohydrates ranging from 45 to 65%. Therefore, at least a portion of our sample consumed relatively high amounts of carbohydrates (60-65%).

Regarding the genotype effects, we found different impacts of the *ABC* genes at the three moments they were analyzed, and two general patterns were observed: some SNPs were associated predominantly with lipid profile at baseline, and with anthropometrics after and in response to the diet. Some of the associations found, especially with the anthropometric variables, were lost after correction for multiple tests. Possible reasons for that are discussed in the study limitations.

Based on the metabolic role of *ABC* genes products, the associations between polymorphisms and baseline lipid profile were expected. We found an additive effect of the *ABCA7* rs2279796 G allele on serum lipids: each G allele added to genotype was associated to an increase in total cholesterol and LDL-c levels. Interestingly, the G allele of this intronic variant was found to increase the *ABCA7* expression in whole blood (Lonsdale *et al.*, 2013), which may alter ABCA7 function and its efficiency in lipid transport. In addition, ABCA7 function is consider “non-essential” to maintenance of body lipids homeostasis. However, ABCA7 could be working in more specific roles, rather than just mimicking ABCA1 (Kim *et al.*, 2005; Abe-Dohmae *et al.*, 2006).

We also found an association between *ABCG1* SNP rs692383 AA genotype and high levels of triglycerides and VLDL. ABCG1 protein function in lipid homeostasis is little known. It is not possible to rule out the possibility that *ABCG1* SNP rs692383 AA genotype influences the gene expression, leading to changes in ABCG1 function and consequently to the cholesterol efflux for HDL enrichment. VLDL particles perform enzymatic exchanges with other lipoproteins, such as HDL (Cheng *et al.*, 2016), thus, this association of *ABCG1* SNP rs692383 AA genotype with VLDL and triglycerides could be a result of changes in HDL role. Interestingly, *ABCG1* SNP rs692383 was the only polymorphism found to be associated with HDL-c levels changes in response to the diet. It seems participants with the AA genotype were more sensitive to the changes promoted by the caloric deficit, in a nutrigenetic mechanism. No other associations between *ABC* genes and HDL-c levels were found and that could be due to the obese state of participants, that *per se* influences in HDL-c levels. In addition, according to Duong *et al.* (2018), obese state impairs the efflux and uptake of cholesterol from hepatic and adipose tissues, in an ABCG1-related mechanism, what agrees with this study findings regarding the difference in LDL-c levels. Summarizing, the AA genotype was associated with a less favorable lipid profile, at baseline and in response to the diet.

At after-diet moment, *ABCG1* SNP rs692383 G allele was associated with higher BMI. In response to diet, *ABCA1* rs1800977 GG genotype, *ABCA1* rs2230806 CC genotype and *ABCG1* rs3827225 A allele were associated with a higher reduction in BMI, waist circumference and abdominal circumference, respectively. These results were not expected at first, since the function of the ABC transporters is believed to impact more on serum lipid levels than on fat stores. However, it is known that there is a strong inverse correlation between obesity and HDL-c levels; especially the central fat storage, that seems to be a predictor of HDL-c levels. The associations found in our study reflects this relationship, since different anthropometric outcomes were associated with *ABC* genotypes which are mainly responsible for modulating the lipid profile (Wang and Peng, 2011; Cheng *et al.*, 2016).

Despite the few studies directly relating the ABC transporters to body adiposity, some relationships can be explored. For example, mice lacking the *ABCA1* gene in adipocytes presented a cholesterol build-up in adipose tissue as a direct effect of a reduction in ABCA1-mediated cholesterol efflux from adipose cells (De Haan *et al.*, 2014; Cuffe *et al.*, 2018). This cholesterol build-up results in an increased adiposity and body weight as a consequence of its influences in the triglycerides storage. On the other hand, Cuffe *et al.* (2018) found that mice lacking the *ABCA1* gene in adipocytes are resistant to diet-induced obesity and have reduced adipose tissue triglycerides storage. Although these studies are controversial, they highlight *ABCA1* gene plays an important role in adipose tissue physiology and can be related with body adiposity management. In addition, *ABCA1* SNP rs1800977 alleles present different expression levels (Frisdal *et al.*, 2015). It is possible that expression levels of *ABCA1* gene are related to cholesterol efflux and this hypothesis is sustained by the linkage disequilibrium (D’ = 0.999) (Lonsdale *et al.*, 2013) existing between the SNP rs1800977 and the functional polymorphism rs9282541 (R230C), whose 230C allele was associated with a reduction of about 27% in cholesterol efflux mediated by ABCA1 (Acuña-Alonzo *et al.*, 2010). This information put the cholesterol metabolism mediated by ABCA1 in context of the resistance to adiposity loss and could be at least one of possible mechanisms responsible for the difference observed between genotypes in weight-loss dietary intervention effectiveness.

The ABCG1 protein seems to have an essential function in adipose tissue, participating in adipocytes lipid storage, mainly in triglycerides storage process (Frisdal *et al.*, 2015). Frisdal *et al.* (2015) found that *ABCG1* gene absence protects mice against diet-induced obesity. The mechanism explained by Frisdal and Le Goff (2015) is that the ABCG1 protein influences the lipoprotein lipase (LPL) function, altering its triacylglycerol-rich lipoproteins hydrolyzation efficiency, leading to altered lipid accumulation in adipocytes. This role meets with the fact that *ABCG1* upregulated expression leads to an increase in the fat mass (Frisdal *et al.*, 2015). This clearly demonstrates that differences in *ABCG1* expression profile could alter lipid deposition in adipose tissue. In addition, obese state itself influences ABC-related cholesterol metabolism: obese mice had impaired reverse cholesterol transportation and lower levels of ABCG1 protein in hepatic and adipose tissues compared to non-obese ones (Edgel *et al.*, 2012). Moreover, calorie restricted obese mice had increased *ABCG1* expression in adipose tissue compared to non-obese mice, and this could be associated with a reduction in the lipid cell content (Edgel *et al.*, 2012). Both studies reveal that the metabolic state could alter the *ABCG1* expression profile in adipose tissue in a nutrigenomic interaction. Therefore, influences of a weight-loss dietary intervention in *ABCG1* expression can be different in individuals with certain *ABCG1* genotypes that *per se* might have a functional effect on the *ABCG1* expression.

Although it has promising results, this study has some limitations, such as a relatively small number of participants and had a high discontinuance rate. Studies with intervention in humans, especially those with changes in lifestyle, are difficult because many participants quit before the conclusion of study, or do not follow the intervention guidelines (Leung *et al.*, 2017). In addition, other intervention studies performed by our group experienced a high drop-out rate by men. Therefore, we chose to work with a sample composed only by women, despite possible hormonal interference in obese-related traits.

Another limitation is missing data about individual age of participants. However, we took care to select only participants older than 20 and who were in the reproductive period of life (premenopausal state), thus, the age range of participants was restricted in a period when sex hormones are active, since the influence of sex hormones on metabolic variables is well known.

Regarding the loss of the significance in many comparisons after multi-test correction, especially in anthropometric variables, this raised the possibility that a longer caloric restriction period, as well as, a higher caloric deficit, would lead to a greater weight reduction and perhaps the genotype-specific metabolic response would be more prominent. This is a possibility, according to many evidences that mainly *ABCA1* and *ABCG1* genes are related with adiposity status. Thus, although some of associations were lost after the p-correction, we choose to discuss some relevant points in this context to stimulate further investigations to clarify these possible associations.

Another limitation was the lack of a functional study to complement our results, leading our discussion to the information available in the literature. The lack of a control group was partially offset with the dietary diary written by the participants to confirm whether diets were properly followed.

To the best of our knowledge, this study is one of the first to investigate the *ABC* gene polymorphisms interaction with a weight-loss dietary intervention in obesity treatment and may have important clinical implications. According to the results, a standard weight-loss diet with a deficit of 600 kcal per day for seven weeks might not be the best option for all obese individuals. However, non-dyslipidemic individuals presenting either *ABCA7* rs2279796 GG genotype and *ABCG1* rs692383 G allele may benefit from this diet. Further investigation is needed to clarify if different genotypes respond differently to specific dietary compositions, allowing the possible identification if either one of the three explored macronutrients – namely, carbohydrate, lipid or protein – has a better compatibility with each genotype. Besides, different caloric deficits and longer intervention periods should be further investigated. Thus, the main strengths of our study are the original results, and the possibility of new investigations to better understand the role of the *ABC* genes in body adiposity.

## Supplementary material

The following online material is available for this article:

Figure S1 Distributions of total cholesterol levels (A) at baseline and after diet and (B) the difference between these two moments in study sample.

Table S1 Allelic and genotypic frequencies.
