# Supplementary material for: Is it possible ABC transporters genetic variants influence the outcomes of a weight-loss diet in obese women?
Source: Genet Mol Biol. 2020 Jul 31;43(3):e20190326. doi: 10.1590/1678-4685-GMB-2019-0326 (PMC7416754; doi:10.1590/1678-4685-GMB-2019-0326)
Supplement: Supplementary file 1 [file 1415-4757-GMB-43-3-e20190326-suppl2.pdf]

Genetics and Molecular Biology
https://doi.org/10.1590/1678-4685-GMB-2019-0326

Supplementary material to "Is it possible ABC transporters genetic variants influence the outcomes of a weight-loss diet in obese women?"

Table S1. Allelic and genotypic frequencies

Gene	SNP	n	Allelic frequency (%)		SE	Genotypic frequency (%)		*P* value
ABCA1	rs1800977	209	A	37.30	0.01	AA	13.40	0.947
			G	62.70		AG	47.80	
						GG	38.80	
	rs2230806	205	C	60.00	0.01	CC	45.80	**< 0.001***
			T	40.00		CT	28.30	
						TT	25.90	
ABCA7	rs2279796	201	A	56.90	0.01	AA	21.40	0.992
			G	43.10		AG	49.25	
						GG	29.35	
ABCG1	rs692383	209	A	56.90	0.01	AA	34.93	0.335
			G	43.10		AG	44.02	
						GG	21.05	
	rs3827225	207	A	22.50	0.01	AA	3.40	0.388
			G	77.50		AG	38.20	
						GG	58.40	

NOTES: *P* values correspond to Hardy-Weinberg equilibrium; SE: standard error. * Comparisons with significant *P* value.
