# Supplementary material for: Is it possible ABC transporters genetic variants influence the outcomes of a weight-loss diet in obese women?
Source: Genet Mol Biol. 2020 Jul 31;43(3):e20190326. doi: 10.1590/1678-4685-GMB-2019-0326 (PMC7416754; doi:10.1590/1678-4685-GMB-2019-0326)
Supplement: Supplementary file 2 [file 1415-4757-GMB-43-3-e20190326-suppl1.pdf]

Genetics and Molecular Biology
https://doi.org/10.1590/1678-4685-GMB-2019-0326

Supplementary material to "Is it possible ABC transporters genetic variants influence the outcomes of a weight-loss diet in obese women?"

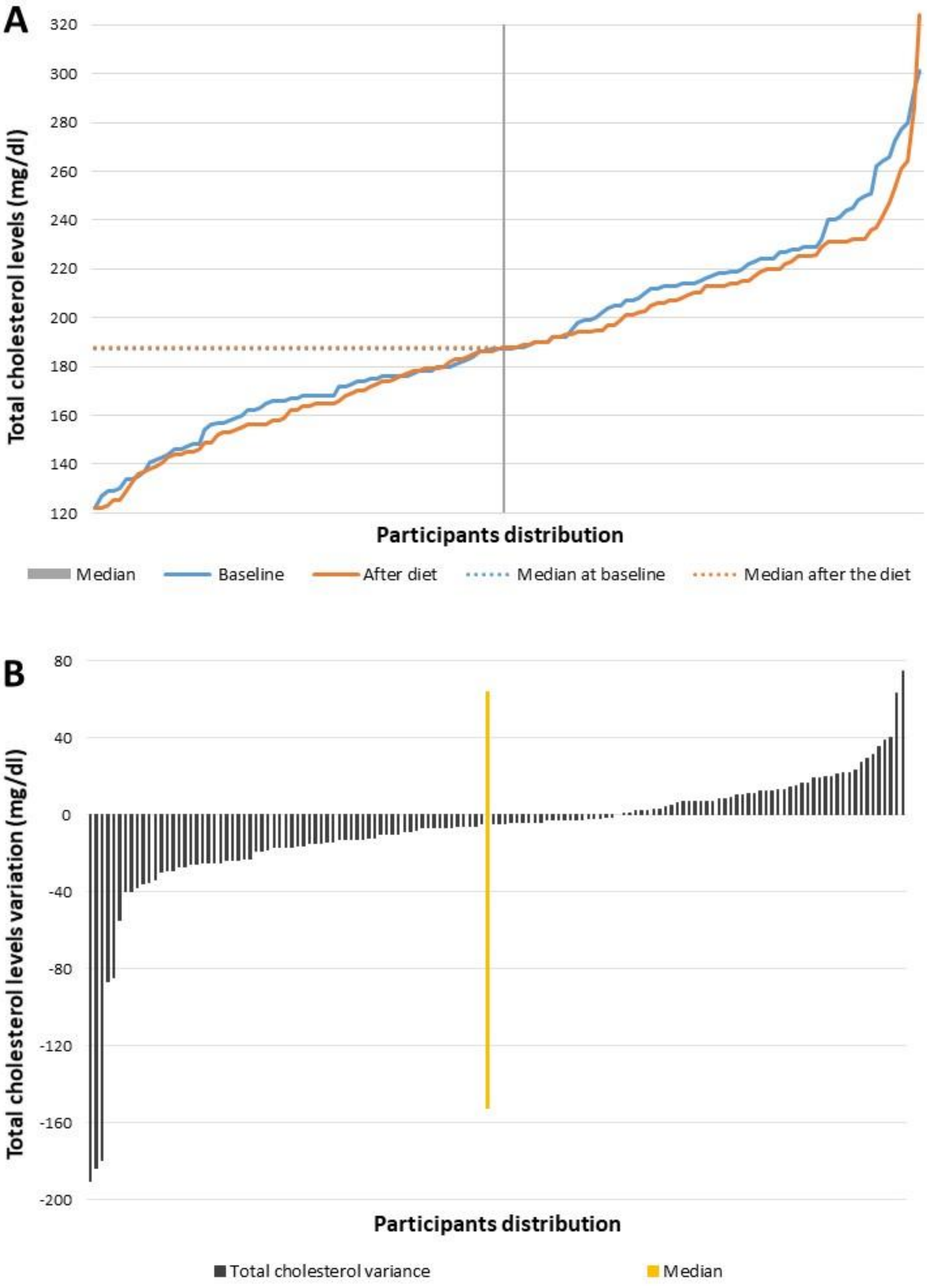

Figure S1. Distributions of total cholesterol levels (A) at baseline and after diet and (B) the difference between these two moments in study sample.
